# Gain-of-function in *Arabidopsis* (GAINA) for identifying functional genes in *Hevea brasiliensis*

**DOI:** 10.1186/s40064-016-3523-4

**Published:** 2016-10-22

**Authors:** Han Cheng, Jing Gao, Haibin Cai, Jianshun Zhu, Huasun Huang

**Affiliations:** 1 Key Laboratory of Ministry of Agriculture for Tropical Crops Physiology, Rubber Research Institute, Chinese Academy of Tropical Agricultural Science, Danzhou City, Hainan People’s Republic of China; 2 Rubber Research Institute, Chinese Academy of Tropical Agricultural Science, Danzhou, 571737 Hainan People’s Republic of China

**Keywords:** Gain-of-function, Rubber tree, *Arabidopsis*, Full-length cDNA library, Mutation, Forward genetics

## Abstract

**Background:**

Forward genetics approaches are not popularly applied in non-model plants due to their complex genomes, long life cycles, backward genetic studies etc. Researchers have to adopt reverse genetic methods to characterize gene functions in non-model plants individually, the efficiency of which is usually low.

**Results:**

In this study, we report a gain-of-function in *Arabidopsis* (GAINA) strategy which can be used for batch identification of functional genes in a plant species. This strategy aims to obtain the gain-of-function of rubber tree genes through overexpressing transformation ready full-length cDNA libraries in *Arabidopsis*. An initial transformation test produced about two thousand independent transgenic *Arabidopsis* lines, in which multiple obvious aberrant phenotypes were observed, suggesting the gain-of-function of rubber tree genes. The transferred genes were further isolated and identified. One gene identified to be metallothionein-like protein type 3 gene was further transferred into *Arabidopsis* and reproduced a similar aberrant phenotype.

**Conclusion:**

The GAINA system proves to be an efficient tool for batch identification of functional genes in *Hevea brasiliensis*, and also applicable in other non-model plants.

**Electronic supplementary material:**

The online version of this article (doi:10.1186/s40064-016-3523-4) contains supplementary material, which is available to authorized users.

## Background

Mutants play important roles for identifying new genes with specific functions. Many methods have been applied to generate loss-of-function mutations, including the use of ethyl methanesulfonate, fast-neutron treatment, antisense and RNA interference technology, and insertion mutations by a transposable element or T-DNA (Bolle et al. 2011). These methods have produced a large number of mutant pools in *Arabidopsis* (Alonso et al. 2003; Berardini et al. 2004; Lamesch et al. 2012), rice (Zhang et al. 2006; Krishnan et al. 2009) and maize (Andorf et al. 2015). However, these approaches do not likely discover the genes that are redundant or essential for early embryo development, as these mutations may cause embryo lethality that gives no offsprings. Gain-of-function strategy works efficiently to overcome these shortcomings, which enhances gene expression to generate mutation phenotypes (Weigel et al. 2000; Nakazawa et al. 2003).

Activation tagging was the first and most widely used gain-of-function mutation method which utilizes the enhancer element from the cauliflower mosaic virus (CaMV) 35S gene. T-DNA containing four folds 35S enhancers is transferred into the *Arabidopsis* genome and activates the nearby gene transcription (Weigel et al. 2000; Nakazawa et al. 2003). Activation tagging now has been successfully applied in rice (Jeong et al. 2002), tomato (Mathews et al. 2003), poplar (Fladung and Polak 2012) etc. A new strategy for activation tagging utilizes a recombinase reaction between two lines generated by the pEnLox/pCre vector system, which provides a new and easier way to analyze gain-of-function mutants (Pogorelko et al. 2008). Another strategy such as SARE (Sense/Antisense RNA Expression) overexpresses genes in sense or antisense manner directly at genome scale, which produces enhanced or suppressed mutants in the same mutant pools (Mou et al. 2002). The SARE system constructes an *Arabidopsis* cDNA library by inserting the cDNA fragments between the 35S promoter and NOS terminator. This expression library is then used to transform *Arabidopsis* through an *Agrobacterium*-mediated way. The initial application of this system has isolated a mutant overexpressing the sense cDNA fragment of a choline biosynthesis-related PEAMT gene (Mou et al. 2002). However, the combination of gain-of-function and loss-of-function mutants in the same pool would increase the difficulty of mutants screening.

All these methods require the homogenization of the mutation allele, and an effective transgenic technique to produce enough transgenic lines for saturating the genome (Peters et al. 2003). However, it is not easy to be achieved in the plants with a long life cycle such as the rubber trees (*Hevea brasiliensis*), which produce seeds at 4 or 5 years old (Priyadarshan and de Goncalves 2003). Besides, rubber trees are highly heterozygous and recalcitrant to transform, let alone to produce plenty of transgenic lines for screening mutations (Montoro et al. 2003; Jayashree et al. 2003; Blanc et al. 2006; Leclercq et al. 2010). So it is not feasible to utilize classical genetic approaches to identify genes in rubber tree. Here we report a new strategy to overexpress rubber tree genes in *Arabidopsis*, which has proved to be fast and effective for the identification of functional gene in *H. brasiliensis*.

## Methods

### Plant materials and growth conditions

*Arabidopsis thaliana* L. Heynh. ecotype Columbia (*Col-0*) was used in this study. Plant growth conditions were described elsewhere (Cheng et al. 2015).

Rubber tree (*H. brasiliensis*) clone 93-114 was used for RNA extraction (Cheng et al. 2008). This genotype was not EST sequenced before this work. The seedlings were grown in a green house with 12 h light/12 h dark photoperiod (120 μE m^−2^ s^−1^). When the seedlings were about 1 m high, with the second whirl leaves stabilized, the leaves and bark were harvested and frosted in liquid nitrogen for RNA extraction.

### Construction of pXCS-LIB binary vector

The expression vector pXCS-LIB was derived from the pXCS-HAStrep (accession number AY457636), which was provided by Dr. Claus-Peter Witte (Witte et al. 2004). To generate pXCS-LIB, an adapter (AAGCTTGGCCATTACGGCCAATAGGCCGCCTCGGCCGAATTC, *Hind*III and *EcoR*I sites underlined) was ligated into the *Hind*III and *EcoR*I site in pXCS-HAStrep. The new constructed plasmid was sequenced using primer LibSeq (5′-TCCTTCGCAAGACCCTTCCT-3′) to confirm right structure. The pXCS-LIB was digested by *Sfi*I, and then dephosphorylated by calf intestinal alkaline phosphatase (Takara). The digested pXCS-LIB fragment was recovered and used for cDNA library construction.

### Construction of a rubber tree cDNA library in pXCS-LIB

Rubber tree total RNA was extracted from mix sample of the leaf, bark and shoot tips by CTAB method (Cheng et al. 2015), and mRNA was isolated using a PolyATract mRNA Isolation System III (Promega). The cDNA was synthesized using the Clontech Creator SMART cDNA Library Construction Kit, and was then normalized with TRIMMER-DIRECT cDNA Normalization Kit (Evrogen). The normalized cDNA was then digested by *Sfi*I and fractioned using CHROMA SPIN-400 Columns. The cDNA longer than 300 bp was recovered and ligated into the *Sfi*I digested pXCS-LIB fragments. The ligation products were ethanol precipitated and dissolved in 5 µL double distilled water, and electroporated into 25 µL *E.coli* TOP10 competent cells using a Gibco BRL Cell Porator with the follow setting: capacitance 330 μF, voltage 350 V, impedance low ohms, charge rate fast, resistance 4 kΩ. After transformation, the cell was resuspended in 1 mL SOC medium and cultured at 37 °C for 45 min. One µL strain culture was diluted into 100 µL LB medium, and plated onto LB agar plate containing 50 mg/L carbenicillin. After overnight culture, the clones were counted and the library titer was calculated.

For cDNA insertion size determination, clones were randomly picked up and subjected to PCR analysis with primer with primer F-p (5′-TCCTTCGCAAGACCCTTCCT-3′) and R-p (5′-TGAGGATGAGACCAACCGGC-3′). The products were resolved on agarose gel and the bands size was further calculated.

### Transfer the binary plasmid library into *Agrobacteria* and generation of transgenic plants

The cDNA library was amplified on two hundreds 15 cm LB agar plates and all the clones were combined in 1000 mL liquid LB medium. The library plasmid was extracted from 100 mL of the amplified cDNA library using a Qiagen plasmid purification kit. This library plasmids were further introduced into *Agrobacterium tumefacien*s strain GV3101 (*pMP90RK*) by electroporation, and transformants were screened on YEB agar plates supplemented with 50 mg/L rifamycin, 20 mg/L gentamicin, 50 mg/L kanamycin, and 50 mg/mL carbenicillin (Koncz and Schell 1986). Transformants were allowed to grow on plates for 3 days and then were pooled and cultured for 2 h at 28 °C.

*Arabidopsis* *Col-0 *plants were transformed with these agrobacteria via flower dipping method (Clough and Bent 1998). Plants prepared for transformation were grown on the medium composed vermiculite and peatmoss (1:3) at 23 °C. Transformed plants were allowed to self-pollination and the seeds were harvested. Transgenic plants were selected by spraying 100 mg/L Basta (glufosinate ammonium) onto 5 d T1 plants for 3 times with 3 d intervals. The survived plants were cultured and the T2 seeds from each T1 plant were harvested individually.

### Mutant screening and isolation of the transferred gene

T1 plants were examined for morphological aberrance. Individual T1 plants with specific phenotype were subjected to further analysis. For each mutant line, segregation of phenotype and Basta resistance was performed in the T2 and T3 generations. The lines with a 3:1 segregating ratio was regarded as single T-DNA insertion.

The overexpressed genes were isolated by amplifying the genomic DNA of the mutants using primers LIB5 (5′-ATGACGCACAATCCCACTATC-3′) and LIB3 (5′-TGTAGAGAGAGACTGGTGATTTTTG-3′). The PCR products were cloned into pMD18-T (Takara) and sequenced.

### Confirmation that the transferred gene leads to the mutant phenotype

To confirm the transferred gene induced the mutant phenotype, the PCR product of target gene was digested by *Sfi*I and ligated into *Sfi*I restrictive sites of pXCS-LIB vector. The constructed plasmid was introduced into *Agrobacterium* strain GV3101 (pMP90RK), and used to transform *Col-0* wild type plants by flower dipping method. The T1 and T2 phenotype of each gene was examined.

### EST sequencing and COG annotation

EST sequencing was conducted with ABI 3730 platform at BGI Company (Beijing, China). The raw sequencing data were first cleansed to get rid of vector sequence, low quality ESTs and chimeric sequences. Then the clean ESTs were assembled with CAP3 program using the parameters: identity, 0.95; minimal overlap, 50 bp (Huang and Madan 1999). The assembled unigenes were then used for BLASTx searches (E-value < 1e-5) and annotation against SWISSPROT, KEGG and COG databases.

### Availability of data and materials

The datasets supporting the conclusions of this article are included within the article and its additional file. The EST sequences were submitted to GenBank as dataset.

## Results

### Overview of the GAINA system

**Fig. 1:**
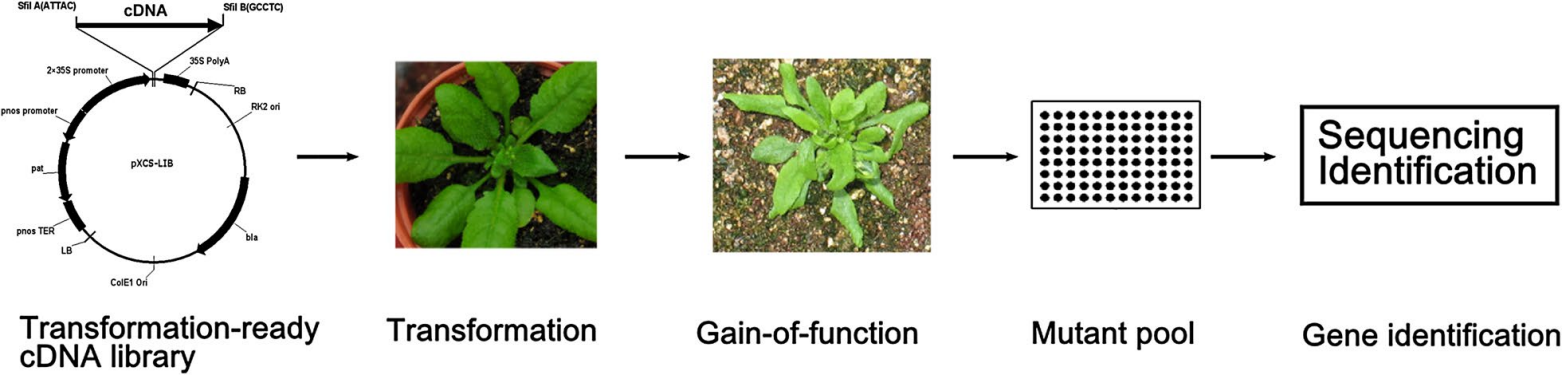
Overview of GAINA system. Workflow of gain-of-function strategy for identification functional genes in rubber tree

**Fig. 2:**
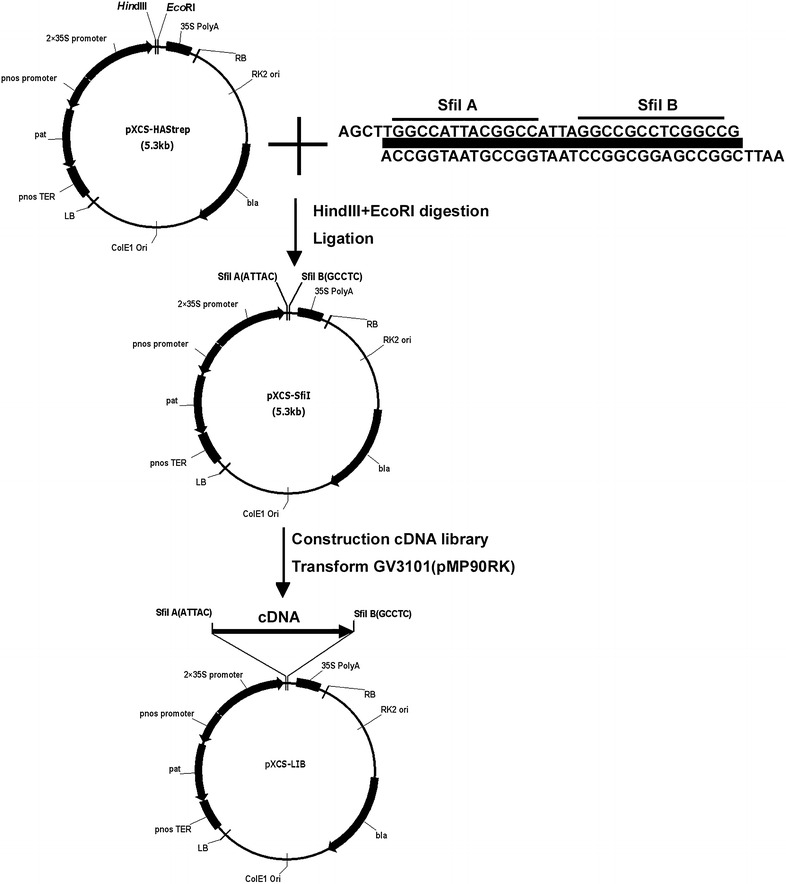
The construction of expressible full-length cDNA library. A adapter with *Sfi*l A and *Sfi*l B restrictive sites was introduced into pXCS-HAStrep to generate binary vector pXCS-lib, which was fully compatible with Clontech Creator SMART cDNA Library Construction Kit. Then the full-length cDNA library was constructed in this vector

The GAINA system overexpress rubber tree genes in *Arabidopsis*, and generate gain-of-function mutant pools for rubber tree genes. The gain-of-function mutant pools are then used for functional gene identification (Fig. 1). To obtain overexpression lines of rubber tree genes, a transgene-ready full length cDNA library was constructed. To fulfill this, we first constructed a binary vector designated as pXCS-LIB that is used for full length cDNA cloning in sense direction and is fully compatible with the Clontech Creator SMART cDNA Library Construction Kit (Fig. 2) (Zhu et al. 2001). The rubber tree full-length cDNAs were then cloned into the *Sfi*l A and *Sfi*l B sites, and resulted in the transgene-ready full length cDNA library. As the full length cDNAs were driven by a double 35S promoter and followed by a 35S poly A terminator, the cloned cDNAs were expressible in plants when transferred as part of the T-DNA fragment. The transformation ready cDNA library was then transformed into *Arabidopsis* in an *Agrobacterium*-mediated flowering dipping method. Independent transgenic lines were collected and each contained at least one overexpressed rubber tree gene. These lines constitute the gain-of-function *Arabidopsis* mutant pools of the rubber tree genome information facilitating functional genes identification in this non-model species.

### Construction of a rubber tree cDNA library

The key point of GAINA system is to construct a high-quality transformation ready cDNA library with uniform abundance and broad representation. To achieve this goal, the RNA was extracted from mixed samples of leaf, bark and shoot tips, then the cDNA was further normalized. Besides, the cDNAs should contain intact CDS in the sense direction which ensures the cloned cDNAs are expressed and translated correctly. To meet these criteria, we constructed a normalized full-length cDNA library using the SMART™ and TRIMMER™ technology (Zhu et al. 2001; Bogdanov et al. 2010). The normalized full-length cDNA fragments were then cloned into the *Sfi*l restrictive site in sense direction and transformed into TOP10 competent cells. An aliquot of the cDNA library is titrated which demonstrated this library contained 1.4 × 10^6^ clones.

**Fig. 3:**
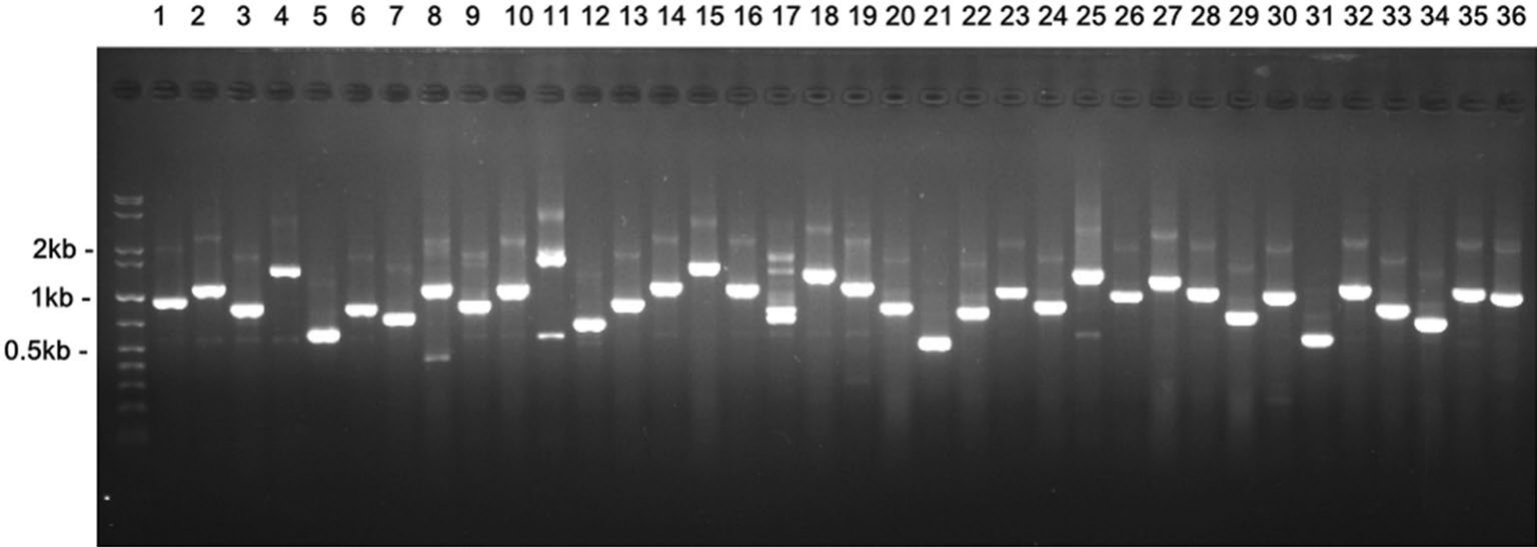
Size distribution of cDNA fragments in constructed full-length cDNA library. Thirty-six random selected clones were subjected to PCR amplification to determine the inserted fragment size

To evaluate the quality, diversity and insertion length of the constructed full length cDNA library, we randomly picked up thirty-six clones and analyzed with PCR amplification. As shown in Fig. 3, all the selected clones contained effective insertions. The cDNA insert length ranged from 0.5 to 2 kb with an average size of 1 kb, demonstrating this library was of high quality and rich diversity.

### EST sequence analysis

The normalized full length cDNA library was subjected to EST sequencing from the 5′ terminal using Sanger sequencing technology. Totally 25020 EST sequences were obtained, of which 22585 were high-quality sequences. Using a CAP3 assembly program (Calikowski and Meier 2006), the ESTs obtained were assembled into 12114 unigenes (3233 contigs and 8881 singlets), with a redundancy rate of about 46 %. The EST sequencing data demonstrated a high quality and wide representation of this library for rubber tree transcriptomes.

**Fig. 4:**
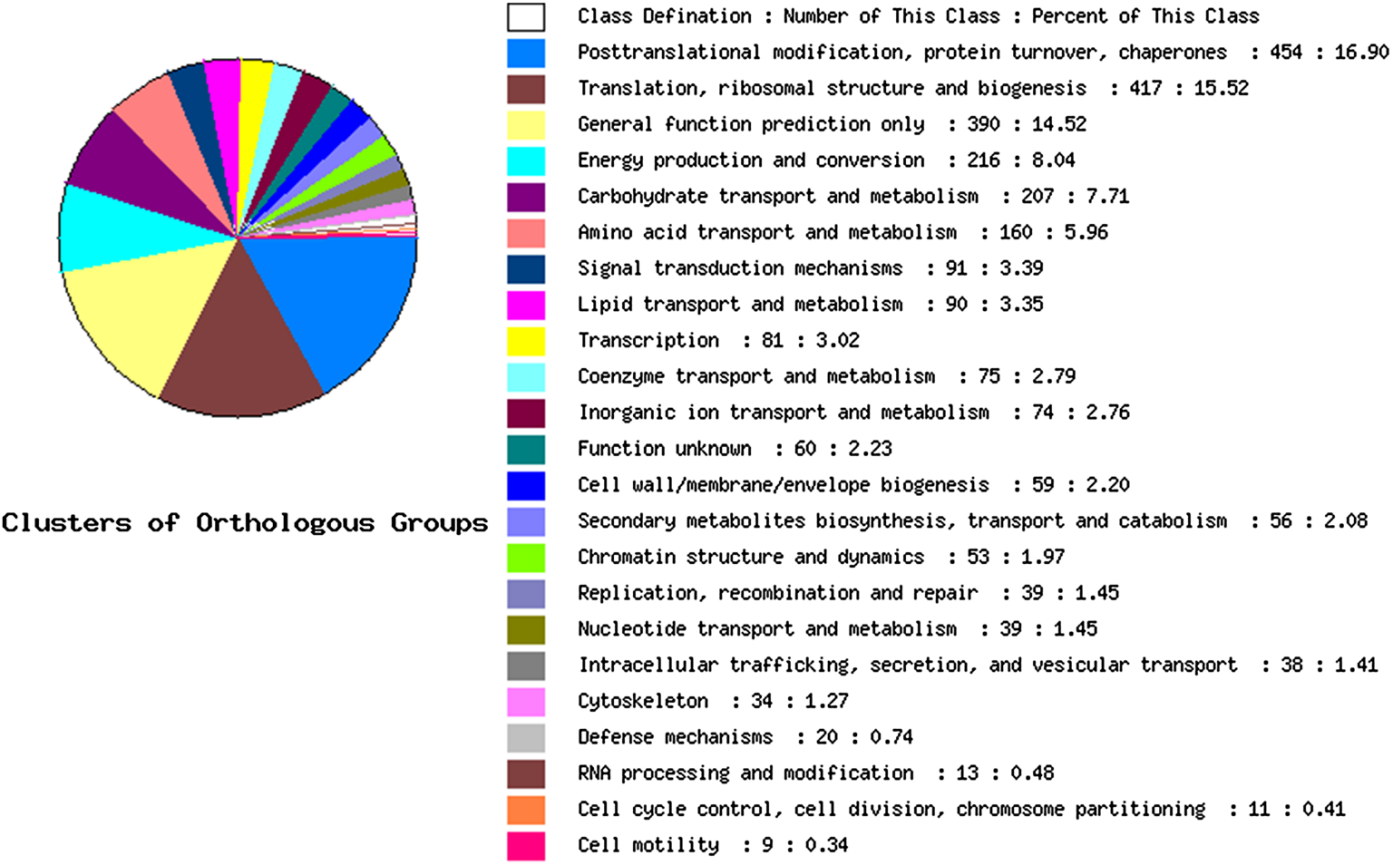
Cluster of orthologous groups annotation profile for unigenes of 25020 sequenced EST. Totally 12114 unigenes were subjected to annotation by blastx program against SWISSPROT, KEGG and COG databases. The classes definition, number of unigenes in each class and the percentage of each class were listed in the *right*

The ESTs sequences were also annotated using blastx against SWISSPROT, KEGG and COG databases. Figure 4 showed a profile of COGs annotation of all unigenes. Totally 3127 genes were annotated with COG database (e-value cut-off 1E-05), belonging to 757 COG ids. These COGs fell into 23 COGs classes, with posttranslational modification (16.9 %), translation (15.5 %) and general function prediction (14.5 %) as the major part. The COGs classes’ diversity of the annotated unigenes demonstrated the library represented genes with versatile functions.

### Multiple gain-of-function lines with visible phenotype aberrance

**Fig. 5:**
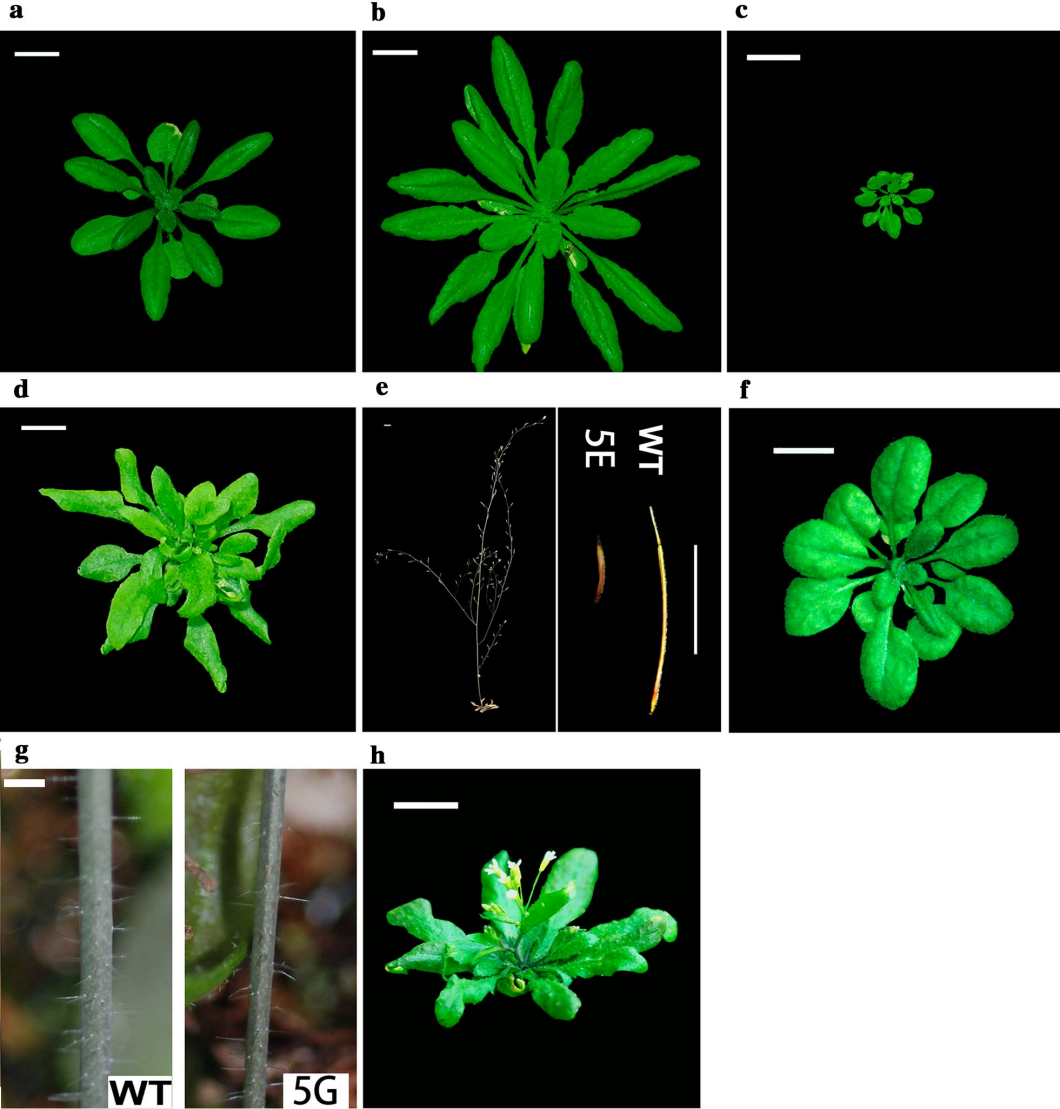
T1 generation lines showed obvious phenotype aberrance. **a** *Col*-*0* seedlings. **b**–**g** Independent transgenic lines show phenotype aberrance. **b** Enlarged rosette size; **c** Small rosette size; **d** Twisted rosette leaves; **e** Short silique; **f** Round rosette leaves; **g** Slim inflorescence and few trichomes. The photos were taken at 22 days after germination for **a**, **b**, **c**, **d**, **f**. *WT* wild type phenotype, *5E* phenotype of 5E mutant, *5G* phenotype of 5G mutant. *Bar* 1 cm

The full length cDNA library was amplified on LB agar plates and the plasmids were extracted and transferred into *Agrobacterium* strain GV3101 (*pMP90RK*). The *Agrobacteria* were then used to transform *Arabidopsis* seedlings with flower-dip method. Each independent overexpression line was selected and regarded as one gain-of-function line of a particular gene. Totally about 2000 independent gain-of-function lines were obtained in an initial round of screening. Among these lines, more than one hundred plants showed obvious phenotype aberrance. As shown in Fig. 5, typical aberrant phenotypes included large-sized rosette leaves, small-sized rosette, twist leaves, short siliques etc. (Fig. 5b–g). The diverse aberrant phenotype of the transgenic lines indicated that the gain-of-function of rubber tree genes could result in *Arabidopsis* phenotype changes and thus be applicable to identify genes of particular interest.

### Isolation of the transferred gene

The inserted rubber tree genes were then cloned from *Arabidopsis* gain-of-function lines using PCR method. Four lines (phenotype 5C to 5F) were characterized, each of which contained only one T-DNA insertion. Then the cloned fragments were subjected to sequencing, and were then annotated. The annotation results are listed in Table 1, in which two of them were genes with known functions, whereas the other two were annotated as unknown function. The sequences of the cloned genes were shown in Additional file 1: Data 1, Additional file 2: Data 2.

**Table 1:** The identification of the inserted genes in the gain-of-function lines in Fig. 5

ID	Phenotype	Annotation	Sequences	Accession number of predicted gene	Species origin	References
Phenotype 5C	Small rosette and leaves	Mitochondrial substrate carrier family protein	Supplemental Data Phenotype 5C	XP_002314452.2	Populus trichocarpa	Tuskan et al. (2006)
Phenotype 5D	Twist rosette leaves	Metallothionein-like protein type 3	Supplemental Data Phenotype 5D	At3g15353	*Arabidopsis*	Hassinen et al. (2009), Benatti et al. (2014)
Phenotype 5E	Short silique	Unknown function	Supplemental Data Phenotype 5E	At1g55160	*Arabidopsis*	Dunkley et al. (2006)
Phenotype 5F	Round leaves, late flower	Unknown function	Supplemental Data Phenotype 5F	OAY30768.1	Manihot esculenta	n.a.

The ID phenotype 5C to phenotype 5F refer to the lines in Fig. 5c–f respectively

To test if overexpression of the rubber tree genes could reproduce the phenotypes found in the gain-of-function mutant pools, the phenotype 5D gene (metallothionein-like protein type 3) was re-overexpressed in *Arabidopsis*. The transgenic seedlings also displayed twisted leaves phenotype as found in gain-of-function mutant pools (Fig. 5h). These results demonstrated that the gain-of-function phenotype found in Fig. 5d did come from the overexpression of the transferred rubber tree metallothionein-like protein type 3 gene.

## Discussion

Chemical and physical mutation is the most frequently used method to generate mutants. Though saturating a genome is relatively easy for chemical and physical mutation, it is difficult to map the mutation site, thus makes it very difficult to clone the target genes. Though some new methods for mapping the mutation emerged recent years, such as TILLING etc. (Till et al. 2003), it is still not easy to work in non-model plants. T-DNA insertion has been proved to be very convenient for the cloning of mutated target genes. However, the loss-of-function strategy makes this method to be limited in model plants. For the less-studied long life span trees, the T-DNA insertion mutation is not easy to be achieved. In non-model plants, overexpression in *Arabidopsis* is an alternative method to characterize the gene functions, which has been successfully applied in many plant species (Foucart et al. 2009; Kalamaki et al. 2009; Jiang et al. 2016). This gain-of-function strategy is very useful for the plants with low transformation efficiency.

In this study, we adopt a gain-of-function method (GAINA) to overexpress the rubber tree full-length cDNA library in *Arabidopsis*, and generate thousands of gain-of-function lines. These lines contain overexpressed rubber tree genes, and therefore can be used to identify genes with particular functions. The key of the GAINA system is to construct a full-length cDNA library in the sense direction, which ensures the successful expression after transferred into *Arabidopsis*. First we constructed a transformation-ready binary vector pXCS-lib which is compatible to full-length cDNA library construction. The full-length cDNA library was then cloned into pXCS-lib plasmid and utilized to transform *Arabidopsis*. To ensure the cDNA library mostly represent the rubber tree genes, a mixed RNA sample from several tissues was used for library construction. A normalization step was further used to gain uniform abundance for each gene in the library. These measures guaranteed the maximum likelihood to obtain gain-of-function mutation lines for each rubber tree genes. The EST sequencing of the full-length cDNA library also confirmed the wide representation of the constructed library.

Another key of the GAINA system is to generate enough *Arabidopsis* transgenic lines, which maximizes the coverage of the rubber tree genes. In the initial experiment, two thousands independent transgenic lines were obtained, in which aberrant phenotypes were observed in many lines. This also demonstrated that the GAINA strategy was effective to identify functional genes in the rubber trees, especially for the genes involved in organ development, abiotic stress resistance. The next job is to obtain more GAINA transgenic lines, and to cover more rubber tree genes in this system.

In this study, four rubber tree genes are identified using GAINA system. The transferring of these genes caused visible phenotype aberrances in *Arabidopsis* (Fig. 5). Among these identified genes, only one (MT3) was previously well characterized. MT3 is low molecular weight, cycteine-rich proteins that bind metals such as Zn, Cu or Cd, and is proposed to participate in a variety of processes including metal ion homeostasis and tolerance (Benatti et al. 2014). *MT3* is also suggested to protect cells against oxidative stress (Akashi et al. 2004). The overexpression of the rubber tree *MT3* gene caused twisted leaves in GAINA system. This phenotype was reproduced in an independent *HbMT3* transgene experiment, which further confirmed the connection between phenotype and *HbMT3* overexpression, though the detail mechanism is yet to be unraveled.

Nowadays, there were numerous studies that transferred the non-model plants genes into *Arabidopsis* and characterized their functions (Foucart et al. 2009; Kalamaki et al. 2009; Polashock et al. 2010; Jiang et al. 2016; Wang et al. 2016; Liu et al. 2016). We also characterized rubber tree *HbCBF1* gene which showed conserved functions in regulating CBF pathway in *Arabidopsis* (Cheng et al. 2015). These studies utilized the reverse genetic approach to characterize gene functions. The GAINA system provides a forward genetic approach for batch identification of functional genes in non-model plants, which will greatly facilitate the genetic study in these species. However, a shortcoming of the GAINA system is that this method will not likely identify rubber tree specific genes, such as those involved in rubber biosynthesis, latex development. The study of these genes should rely on the molecular biology research progresses in *H. brasiliensis* (Kush et al. 1990; Duan et al. 2010; Chow et al. 2012). Even so, the GAINA system offers a powerful forward genetic tool for gene studies, which will greatly help researchers to identify genes that involved in particular functions in non-model plant species.

## Conclusion

In this study, we report a gain-of-function in *Arabidopsis* (GAINA) systemthat overexpresses a rubber tree full-length cDNA library in *Arabidopsis*, and generates thousands of transgenic lines. These gain-of-function lines prove to be beneficial for the functional identification of rubber tree genes. Therefore, the GAINA system offers a powerful forward genetic tool for functional gene study and the identification of genes involved in particular pathways in non-model plant species.

## Additional files

**Additional file 1: Data 1.** The sequences of the cloned genes, phenotype 5C, phenotype 5D, phenotype 5E, phenotype 5F, from the plants of C, D, E, F in Figure 5.

**Additional file 2: Data 2.** Blastx data of the cloned genes, phenotype 5C, phenotype 5D, phenotype 5E, phenotype 5F, from the plants of C, D, E, F in Figure 5.

## Abbreviations

GAINA: gain-of-function in *Arabidopsis*
MT3: metallothionein-like protein type 3

## References

[CR1] Akashi K, Nishimura N, Ishida Y, Yokota A (2004). Potent hydroxyl radical-scavenging activity of drought-induced type-2 metallothionein in wild watermelon. Biochem Biophys Res Commun.

[CR2] Alonso JM, Stepanova AN, Leisse TJ (2003). Genome-wide insertional mutagenesis of *Arabidopsis thaliana*. Science.

[CR3] Andorf CM, Cannon EK, Portwood JL et al (2015) MaizeGDB update: new tools, data and interface for the maize model organism database. Nucleic Acids Res gkv1007. doi:10.1093/nar/gkv1007 10.1093/nar/gkv1007 PMC4702771 26432828

[CR4] Benatti R, Yookongkaew N, Meetam M (2014). Metallothionein deficiency impacts copper accumulation and redistribution in leaves and seeds of *Arabidopsis*. New Phytol.

[CR5] Berardini TZ, Mundodi S, Reiser L (2004). Functional annotation of the *Arabidopsis* genome using controlled vocabularies. Plant Physiol.

[CR6] Blanc G, Baptiste C, Oliver G (2006). Efficient Agrobacterium tumefaciens-mediated transformation of embryogenic calli and regeneration of *Hevea brasiliensis* Müll Arg. plants. Plant Cell Rep.

[CR7] Bogdanov EA, Shagina I, Barsova EV (2010). Normalizing cDNA libraries. Curr Protoc Mol Biol.

[CR8] Bolle C, Schneider A, Leister D (2011). Perspectives on systematic analyses of gene function in *Arabidopsis thaliana*: new tools, topics and trends. Curr Genom.

[CR9] Calikowski TT, Meier I (2006). Isolation of nuclear proteins. *Arabidopsis* protocols.

[CR10] Cheng H, Cai H, Huang H (2008). Construction of full-length cDNA library in rubber tree under cold stress. Chin J Trop Crops.

[CR11] Cheng H, Cai H, Fu H (2015). Functional characterization of *Hevea brasiliensis* CRT/DRE binding factor 1 gene revealed regulation potential in the CBF pathway of tropical perennial tree. PLoS ONE.

[CR12] Chow K-S, Mat-Isa M-N, Bahari A (2012). Metabolic routes affecting rubber biosynthesis in *Hevea brasiliensis* latex. J Exp Bot.

[CR13] Clough SJ, Bent AF (1998). Floral dip: a simplified method for Agrobacterium-mediated transformation of *Arabidopsis thaliana*. Plant J.

[CR14] Duan C, Rio M, Leclercq J (2010). Gene expression pattern in response to wounding, methyl jasmonate and ethylene in the bark of *Hevea brasiliensis*. Tree Physiol.

[CR15] Dunkley TPJ, Hester S, Shadforth IP (2006). Mapping the *Arabidopsis* organelle proteome. Proc Natl Acad Sci.

[CR16] Fladung M, Polak O (2012). Ac/Ds-transposon activation tagging in poplar: a powerful tool for gene discovery. BMC Genom.

[CR17] Foucart C, Jauneau A, Gion J-M (2009). Overexpression of EgROP1, a Eucalyptus vascular-expressed Rac-like small GTPase, affects secondary xylem formation in *Arabidopsis thaliana*. New Phytol.

[CR18] Hassinen VH, Tuomainen M, Peräniemi S (2009). Metallothioneins 2 and 3 contribute to the metal-adapted phenotype but are not directly linked to Zn accumulation in the metal hyperaccumulator, *Thlaspi caerulescens*. J Exp Bot.

[CR19] Huang X, Madan A (1999). CAP3: a DNA sequence assembly program. Genome Res.

[CR20] Jayashree R, Rekha K, Venkatachalam P (2003). Genetic transformation and regeneration of rubber tree (*Hevea brasiliensis* Muell. Arg) transgenic plants with a constitutive version of an anti-oxidative stress superoxide dismutase gene. Plant Cell Rep.

[CR21] Jeong D-H, An S, Kang H-G (2002). T-DNA insertional mutagenesis for activation tagging in rice. Plant Physiol.

[CR22] Jiang Y, Guo L, Liu R (2016). Overexpression of poplar PtrWRKY89 in transgenic *Arabidopsis* leads to a reduction of disease resistance by regulating defense-related genes in salicylate- and jasmonate-dependent signaling. PLoS ONE.

[CR23] Kalamaki MS, Alexandrou D, Lazari D (2009). Over-expression of a tomato N-acetyl-L-glutamate synthase gene (SlNAGS1) in *Arabidopsis thaliana* results in high ornithine levels and increased tolerance in salt and drought stresses. J Exp Bot.

[CR24] Koncz C, Schell J (1986). The promoter of TL-DNA gene 5 controls the tissue-specific expression of chimaeric genes carried by a novel type of Agrobacterium binary vector. Mol Gen Genet MGG.

[CR25] Krishnan A, Guiderdoni E, An G (2009). Mutant Resources in Rice for Functional Genomics of the Grasses. Plant Physiol.

[CR26] Kush A, Goyvaerts E, Chye ML, Chua NH (1990). Laticifer-specific gene expression in *Hevea brasiliensis* (rubber tree). Proc Natl Acad Sci.

[CR27] Lamesch P, Berardini TZ, Li D (2012). The *Arabidopsis* information resource (TAIR): improved gene annotation and new tools. Nucleic Acids Res.

[CR28] Leclercq J, Lardet L, Martin F (2010). The green fluorescent protein as an efficient selection marker for Agrobacterium tumefaciens-mediated transformation in *Hevea brasiliensis* (Müll. Arg). Plant Cell Rep.

[CR29] Liu Y, Yang S, Song Y (2016). Gain-of-function analysis of poplar CLE genes in *Arabidopsis* by exogenous application and over-expression assays. J Exp Bot.

[CR30] Mathews H, Clendennen SK, Caldwell CG (2003). Activation tagging in tomato identifies a transcriptional regulator of anthocyanin biosynthesis, modification, and transport. Plant Cell.

[CR31] Montoro P, Rattana W, Pujade-Renaud V (2003). Production of *Hevea brasiliensis* transgenic embryogenic callus lines by *Agrobacterium tumefaciens*: roles of calcium. Plant Cell Rep.

[CR32] Mou Z, Wang X, Fu Z (2002). Silencing of phosphoethanolamine N-methyltransferase results in temperature-sensitive male sterility and salt hypersensitivity in *Arabidopsis*. Plant Cell.

[CR33] Nakazawa M, Ichikawa T, Ishikawa A (2003). Activation tagging, a novel tool to dissect the functions of a gene family. Plant J Cell Mol Biol.

[CR34] Peters JL, Cnudde F, Gerats T (2003). Forward genetics and map-based cloning approaches. Trends Plant Sci.

[CR35] Pogorelko GV, Fursova OV, Ogarkova OA, Tarasov VA (2008). A new technique for activation tagging in *Arabidopsis*. Gene.

[CR36] Polashock JJ, Arora R, Peng Y (2010). Functional identification of a C-repeat binding factor transcriptional activator from blueberry associated with cold acclimation and freezing tolerance. J Am Soc Hortic Sci.

[CR37] Priyadarshan PM, de Goncalves PS (2003). Hevea gene pool for breeding. Genet Resour Crop Evol.

[CR38] Till BJ, Colbert T, Tompa R (2003). High-throughput TILLING for functional genomics. Methods Mol Biol Clifton NJ.

[CR39] Tuskan GA, Difazio S, Jansson S (2006). The genome of black cottonwood, Populus trichocarpa (Torr. & Gray). Science.

[CR40] Wang T, Sui Z, Liu X (2016). Ectopic expression of a maize hybrid up-regulated gene, ErbB-3 binding Protein 1 (ZmEBP1), increases organ size by promoting cell proliferation in *Arabidopsis*. Plant Sci.

[CR41] Weigel D, Ahn JH, Blázquez MA (2000). Activation tagging in *Arabidopsis*. Plant Physiol.

[CR42] Witte C-P, Noel L, Gielbert J (2004). Rapid one-step protein purification from plant material using the eight-amino acid StrepII epitope. Plant Mol Biol.

[CR43] Zhang J, Li C, Wu C (2006). RMD: a rice mutant database for functional analysis of the rice genome. Nucleic Acids Res.

[CR44] Zhu YY, Machleder EM, Chenchik A (2001). Reverse transcriptase template switching: a SMART approach for full-length cDNA library construction. Biotechniques.

